# Fecal Microbiota of Toxigenic *Clostridioides difficile*-Associated Diarrhea

**DOI:** 10.3389/fmicb.2018.03331

**Published:** 2019-01-14

**Authors:** Marta Hernández, Mónica de Frutos, David Rodríguez-Lázaro, Luis López-Urrutia, Narciso M. Quijada, Jose María Eiros

**Affiliations:** ^1^Laboratorio de Biología Molecular y Microbiología, Instituto Tecnológico Agrario de Castilla y León, Valladolid, Spain; ^2^Área de Microbiología, Departamento de Biotecnología y Ciencia de los Alimentos, Universidad de Burgos, Burgos, Spain; ^3^Hospital Universitario Río Hortega, Valladolid, Spain

**Keywords:** CDI, *Clostridioides difficile*, diarrhea, microbiota, bacterial, 16S rRNA

## Abstract

*Clostridioides difficile* infection (CDI) is currently one of the most important causes of infectious diarrhea in developed countries and the main cause in healthcare settings. Here, we characterized the gut microbiota from the feces of 57 patients with diarrhea from nosocomial and community-acquired CDI. We performed an ecological analysis by high-throughput sequencing of the V3-V4 region of 16S rRNA amplicons and evaluated the association of the various ecological profiles with CDI risk factors. Among all samples *Bacteroidaceae* 31.01%, *Enterobacteriaceae* 9.82%, *Lachnospiraceae* 9.33%, *Tannerellaceae* 6,16%, and *Ruminococcaceae* 5.64%, were the most abundant families. A reduced abundance of *Bacteroides* was associated with a poor CDI prognosis, with severe diarrhea and a high incidence of recurrence. This reduction was associated with a weakened host immune system and previous aggressive antibiotherapy. *Peptostreptococcaceae* family was 1.56% overall and within the family the only identified member was the genus *Clostridioides*, positively correlated with the presence of *Akkermansia* that may be predictive of the presence of a CDI. Finally, a relevant aspect that must be considered in clinical practice is the misdiagnosis of CDI, as patients with a stool sample that tests positive for *C. difficile* are usually diagnosed with CDI and subsequently treated as such. However, co-infection with other pathogenic agents often plays an important role in the development of diarrhea, and must be considered when prescribing antibiotic treatment.

## Introduction

The Gram-positive, spore-forming anaerobe, *Clostridioides difficile* (formerly *Clostridium difficile* and *Peptoclostridium difficile*; Yutin and Galperin, 2013) is an asymptomatic component of the healthy intestinal microbiota of approximately 2–7% of healthy human adults and up to 70% of healthy newborns (McFarland et al., 1989; Lees et al., 2016). However, certain *C. difficile* strains have pathogenic potential mediated by two exotoxins: toxin A (TcdA) and toxin B (TcdB), encoded by the *tcd*A and *tcd*B genes within the pathogenicity loci (PaLoc). In addition, some *C. difficile* strains may also produce a binary toxin, called *C. difficile* transferase (CDT), with a potential role in the pathogenesis of the bacterium (Di Bella et al., 2016). *C. difficile* infection (CDI) is thus a toxin-mediated disease of the colon, with clinical symptoms that range from mild or self-limiting diarrhea to pseudomembranous colitis and life-threatening fulminant colitis (Leffler and Lamont, 2015; Smits et al., 2016).

*Clostridium difficile* infection is currently one of the most important causes of infectious diarrhea in developed countries and the main cause in healthcare settings (Lagier, 2016). The rate of recurrence and mortality has been increasing since 2002, associated with severe infections produced by the emergence of the hyper-virulent ribotype 027 (ST1) strain. The burden of CDI has increased mainly in the United States and Europe. It is estimated that nearly 500,000 illnesses and 15,000 deaths are caused by CDI every year in the United States ^1^, whereas the annual incidence in the EU was estimated to be 123,997 cases in 2011–2012^2^, with mortality rates of 3–30% (Hensgens et al., 2013). Remarkably, although 125 ribotypes have being found in Europe, the hyper-virulent ribotype 027 is the most prevalent (19%) (Davies et al., 2016). In addition, the incidence of CDI is increasing and may be highly underestimated (Alcalá et al., 2015), 23% of cases may be undiagnosed, equivalent to approximately 40,000 missed CDI diagnoses per year in 482 participating hospitals in 20 European countries (Davies et al., 2014). Other relevant factors associated with the emergence of CDI have been the introduction of *C. difficile* strains resistant to multiple antibiotics, including metronidazole, as well as genomic plasticity and the potential to transfer resistance genes, as approximately 11% of the *C. difficile* genome consists of mobile genetic elements (Sebaihia et al., 2006).

*Clostridium difficile* infection appears particularly after antibiotic chemotherapy or prolonged periods of hospitalization, which causes disruption and dysbiosis of the endogenous intestinal microbiota and facilitates the proliferation of toxigenic *C. difficile* in the gut. Fecal-oral transmission from other patients or animals, which represent potential reservoirs of *C. difficile*, also plays an important role in CDI epidemiology (Rupnik, 2007). There are also other risk factors that trigger CDI, including comorbidities, surgical and non-surgical gastrointestinal procedures, admission to an intensive care unit (ICU), an immunocompromised status, and advanced age (>65 years) (Knight et al., 2015). CDI has a high relapse rate due to reactivation or reinfection, making it difficult to completely resolve. However, fecal microbiota transplantation appears to be a promising treatment for recurrent CDI (Shankar et al., 2014; Juul et al., 2018).

It is evident that modification of the gut microbiota can play a relevant role in the development of CDI. The identification of microbial markers that can predict disease severity or chronicity could help in the treatment of patients. Here, we characterized the gut microbiota from the feces of 57 patients with diarrhea from nosocomial or community-acquired CDI. The study consisted of an ecological analysis by high-throughput sequencing of the V3-V4 region of the 16S rRNA amplicons and evaluation of the association of the various ecological profiles with CDI risk factors.

## Materials and Methods

### Sampling and Detection of Toxins

This study was conducted at the Hospital Universitario “Rio Hortega” (Valladolid, Castilla y León, Spain) in accordance with the recommendations of the Ethical Clinical Research Committee (CEIC) of the western health area of Valladolid. The protocol was approved by the CEIC with reference number CEIm PI128-18. All subjects gave written informed consent in accordance with the Declaration of Helsinki.

Fecal content from 57 adults (between 29 and 94 years old, 57/43 male/female ratio) was sampled from November 2016 to April 2018: 51 patients were sampled once, four twice, and two individuals three times. Stool samples were stored at −80°C until use.

Stool samples were initially tested for the presence of both glutamate dehydrogenase (GDH) and toxins A and B by the lateral flow assay *C.Diff Quik Chek Complete assay* (Techlab). The GeneXpert *C. difficile* PCR assay (Cepheid) was used for the detection of the toxin B gene (*tcdB*), binary toxin, and *tcdC* deletion that identifies ribotype 027.

### Measurement of Redox Potential

One gram of feces were diluted in 25 mL of distilled water and centrifuged at 8000 rpm for 10 min. A pH and redox meter (GLP21 and Hach 5262PCE-228-R) was used to measure the pH and the redox potential according to the manufacturer’s instructions.

### Total DNA Extraction

For each stool sample, 220 mg of feces was homogenized and total DNA extracted using the QIAamp DNA Stool Mini Kit (Qiagen), according to manufacturer’s instructions. The DNA concentration was determined using a Qubit^®^fluorimeter (Invitrogen). A second DNA extraction from some of the samples and subsequent sequencing validated the characterization of the microbiota (data not shown).

### 16S rRNA Gene Amplicon Library Preparation and Sequencing

Microbial diversity was studied by sequencing the amplified V3-V4 region of the 16S rRNA gene using previously reported primers and PCR conditions (Klindworth et al., 2013). Sample multiplexing, library purification, and sequencing were carried out as described in the “16S Metagenomic Sequencing Library Preparation” guide by Illumina. Libraries were sequenced on a MiSeq platform at the University of Burgos, leading to 300-bp, paired-end reads.

### Bioinformatics and Data Analysis

Demultiplexed paired-end fastq files were processed using QIIME2 pipeline version 2018.6 (Caporaso et al., 2010) and dada2 (Callahan et al., 2016) and feature-table (McDonald et al., 2012) plugins were used for quality filtering of the reads, merging of the paired ends, chimera removal, and assignation of amplicon sequence variants (ASV). We truncated reverse reads to 240 bp using the “–p-trunc-len-r” option implemented in the dada2 plugin due to decreased quality scores of the sequences at the end of the reverse reads. A phylogenetic tree was built using alignment (Katoh and Standley, 2013) and phylogeny (Price et al., 2010) plugins. Alpha and beta diversity analysis were performed using the diversity^3^ and emperor (Vazquez-Baeza et al., 2013) plugins. Samples were rarefied to 17,520 reads per sample for beta-diversity analysis to reduce the bias due to different sequencing depths (only sample MS1498 was excluded from beta-diversity analysis). A pre-trained Naïve Bayes classifier based on the SILVA database (Pruesse et al., 2007), which had been trimmed to harbor the V3-V4 region of the 16S rRNA gene, was applied to assign taxonomy to the ASV using the feature-classifier plugin (Bokulich et al., 2018).

Plotting was carried out in the R environment^4^, using ggplot2 (Wickham, 2016) and reshape2 (Wickham, 2007) packages. Weighted UniFrac distance matrices calculated with QIIME2 were represented as principal coordinates (principal component analysis - PCoA) to compare bacterial community composition based on the relative abundance of ASV. ASV assigned to the family *Peptostreptococcaceae* were extracted from the feature table and used to construct a heatmap using JColorGrid (Joachimiak et al., 2006).

## Results

### Description of the Study Population

**FIGURE 1:**
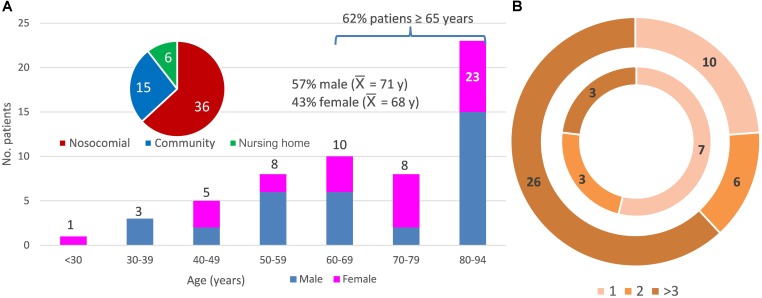
Metadata of the 57 patients studied. **(A)** Distribution of the patients according to age (y, years and 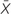, average age), sex (female and male), and community-, healthcare in a nursing home-, and nosocomial-acquired diarrhea. **(B)** Distribution of the patients according to the number of previous hospital admissions (1, 2, or more than 3 previous hospital admissions). The inner ring indicates a community origin (there were two patients with no previous admission not shown in the figure) and the outer ring, nosocomial or healthcare-in-a-nursing home origin of the infection.

Sixty-five fecal samples of the 57 patients with diarrhea enrolled in this study were studied. Figure 1A summarizes the patient metadata; there were no significant differences between gender, 62% of the patients were ≥65 years old, 73.7% were healthcare-associated cases, and 19 patients (33.3%) died before August 2018. All patients were reported to be CDI positive as they were positive for GDH antigen and the gene encoding toxin B (*tcdB*), but negative for the *C. difficile* ribotype 027 test. Some samples were negative for the *in situ* detection of the toxin in the stool and 14 samples were PCR positive for the binary toxin gene (MS0138, MS0148, MS0151, MS0155, MS0211, MS0212, MS0220, MS0223, MS1497, MS1506, MS1508, MS1509, MS1748, MS1753). Remarkably, patients who tested positive for the binary toxin gene did not show severe diarrhea. At least one previous hospital admission was recorded for all but two patients, prior to CDI during the previous year. The number of previous hospital admissions was higher for patients receiving healthcare in hospital than those receiving community care (Figure 1B). All individuals had received antibiotic treatment prior to developing diarrhea and some had also received proton pump inhibitors, except patient MS0215, who came to the emergency room with severe diarrhea, without previous antibiotic treatment. This patient showed an abnormal relative abundance of the *Streptococcus* genus (10.95%) and the *Bacteroides* abundance was below 50%. Upon hospital admission, all inpatients were administrated antibiotics after the positive diagnosis of *C. difficile*. Antibiotherapy generally consisted of metronidazole and then vancomycin if the diarrhea persisted. Only patient MS1496 was finally successfully treated with fidaxomicin, because of multiple recurrences. The most abundant diarrhea-related bacteria for two patients (MS0141 and MS0227) were *Staphylococcus* and *Klebsiella.* Neither co-infection nor these pathogens being the primary cause of diarrhea, instead of *C. difficile*, can be ruled out for these two patients.

### Diversity of Bacterial Microbiota in Feces From Individuals With CDI

A total of 7,474,887 reads survived the quality filtering process (108,239 ± 53,034 reads/sample). This is the first time that QIIME2 has been used to analyze the gut microbiota associated with CDI, and, different from previous studies, sequences are not clustered together into “Operational Taxonomic Units” (OTUs) using a certain dissimilarity threshold (generally 97 or 99% similarity). Amplicon Sequence Variants (ASVs) were obtained instead and represent much higher taxonomic resolution than OTUs, as single-nucleotide differences over the sequenced gene region are taken into account (Callahan et al., 2017). A total of 3,477 ASVs were identified among all samples. We observed lower bacterial diversity than that obtained using OTUs; between 32 (MS0218) and 352 (MS0138) different bacterial ASVs were identified in each fecal sample, significantly lower than the expected 1,000 OTUs that are estimated to exist in a healthy human gut. This reduction of diversity could be associated with the bacterial dysbiosis linked to CDI. However, the main alpha diversity indices, such as the Chao richness estimator, and the Shannon and Simpson diversity indices were 134.32 ± 80.6, 4.01 ± 1.25, and 0.86 ± 0.12, respectively, indicating that the observed reduced alpha diversity was true, and not a sequencing artifact.

**FIGURE 2:**
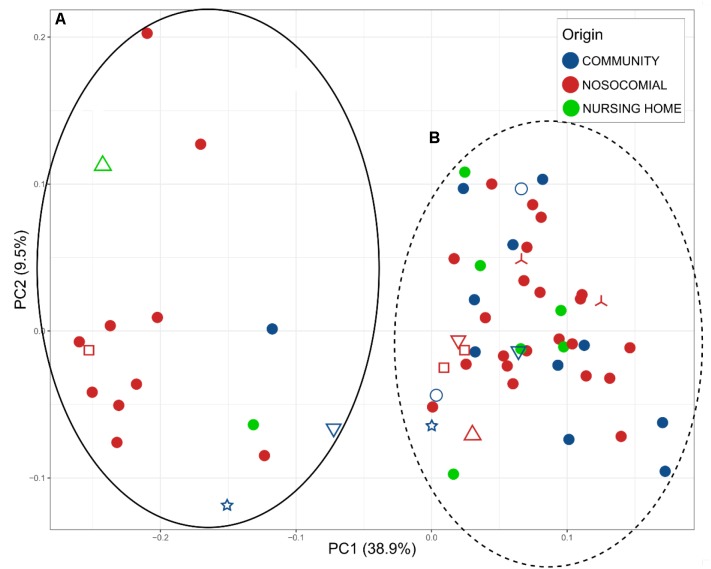
Principal Coordinates Analysis (PCoA), representing the total microbiota of the samples plotted according to the origin of the infection: blue, community; red, nosocomial; and green, nursing home. Two clusters **A** (full black) and **B** (dot lane) were observed. Different samples from the same individual are indicated by the shapes 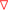
MS0217, MS1502, and MS1193; 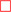 MS0209, MS0222, and MS0214; 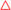 MS0155 and MS0147; 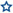 MS01508 and MS1506; 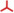 MS0144 and MS0150; and 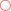 MS1746 and MS1496.

We analyzed beta-diversity between samples by calculating weighted UniFrac distance matrices and representing them as principal coordinates (Figure 2). There were two clusters (A and B) that described 38.9% of the variability in the x-axis. The differences between the two clusters were due to the most abundant bacterial families: *Enterobacteriaceae* and *Enterococcaceae* in cluster A, *Bacteroidaceae* and *Lachnospiraceae* in cluster B. Samples of only 15 patients were assigned to Cluster A (representing 26.31% of the patients). However, given that *C. difficile* could be ruled out as the primary etiological agent for four patients, the percentage dropped to 20.75%. In addition, the PCoA plot showed the points to be more widely dispersed in cluster A than in cluster B (Figure 2). Most of the patients in this cluster were either immunosuppressed (transplant patients) or immunocompromised (cancer patients), with a history of aggressive antibiotherapy. In contrast, cluster B was compact (Figure 2) and contained most of the samples (79.24% of the patients), suggesting that this group may represent the general CDI scenario.

**FIGURE 3:**
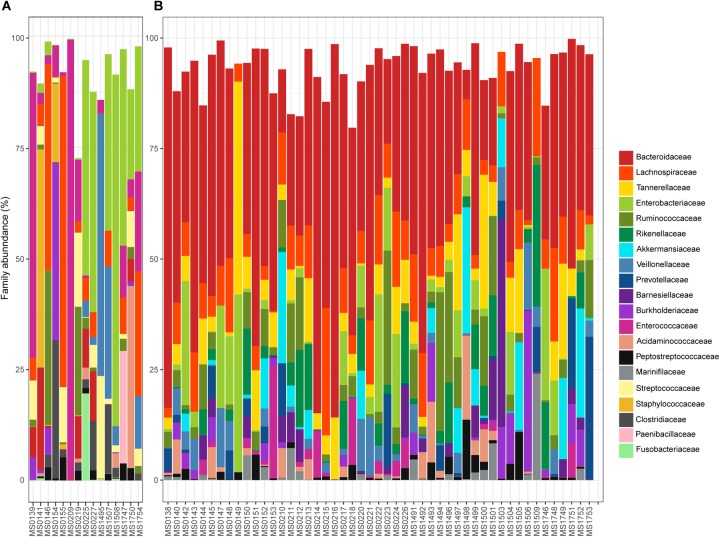
Relative abundance (%) of the 15 most abundant families found in the samples plotted in the **A** or **B** cluster of the PCoA: **(A,B)**, respectively.

The relative abundance of the 15 most abundant families in the fecal samples is shown in Figure 3, in which samples are segregated into clusters A and B from the PCoA. *Bacteroidaceae* was the most abundant family overall (31.01%). However, the percentage varied from 0% in 12 samples to 81.47% in a sample from a patient who had a dental procedure, was treated with clindamycin, and developed mild diarrhea. Patients who lacked *Bacteroidaceae* (mainly in Cluster A) had severe disease and were previously treated for diarrhea with an aggressive or prolonged antimicrobial treatment. In contrast, the diarrhea was less severe and the patients who provided samples in which *Bacteroidaceae* was the most abundant family (mainly Cluster B) recovered easily. Indeed, there was a significant difference in the distribution of *Bacteroidaceae* between cluster A and cluster B; the relative abundance of *Bacteroidaceae* was very low and even absent in many samples (5.57% overall, Figure 3A) in cluster A, whereas it was dominant in cluster B, with a relative abundance of 36.91% (Figure 3B).

Four other bacterial families had an overall relative abundance of over 5%: *Enterobacteriaceae* 9.82%, *Lachnospiraceae* 9.33%, *Tannerellaceae* 6.16%, and *Ruminococcaceae* 5.64%. Together with *Bacteroidaceae*, these five bacterial families represented almost two thirds of the bacterial diversity. Most are obligate anaerobes, indicating severe changes in the redox potential that can produce gut bacterial dysbiosis. Differences in the relative abundance of these bacterial families in clusters A and B are shown in Figure 3. *Enterobacteriaceae* was the most abundant family in cluster A (22.66% overall, but ranged from 0% in sample MS1495 to 79.13% in sample MS1508), whereas the relative abundance of *Lachnospiraceae* was significantly lower (4.96% overall).

Several minority bacterial families (with an overall relative abundance below 5%), such as *Enterococcaceae*, *Veillonellaceae*, *Rikenellaceae*, *Akkermansiaceae*, *Burkholderiaceae*, *Acidaminococcaceae*, *Prevotellaceae*, *Streptococcaceae*, *Barnesiellaceae*, *Peptostreptococcaceae*, *Staphylococcaceae*, *Marinifilaceae*, and *Fusobacteriaceae*, were particularly abundant in some samples. For example, the microbiota of sample MS0227, that came from a patient who had bloody diarrhea, fever, and was vomiting, was dominated by *Enterobacteriaceae* and *Pasteurellaceae* (49.57 and 9.52% relative abundance, respectively) and had a low abundance of *Bacteroidaceae* (11.11%).

**FIGURE 4:**
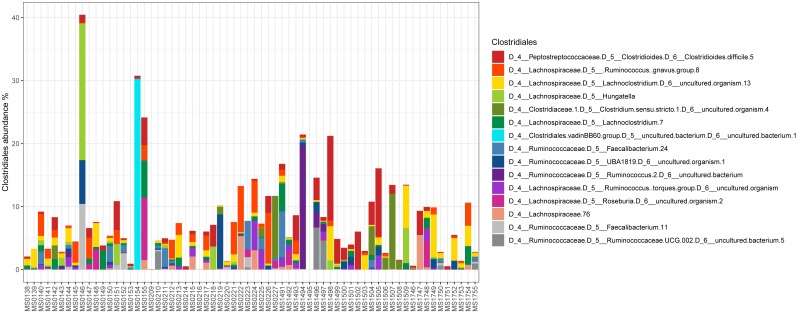
Abundance of taxa within the order *Clostridiales*.

**FIGURE 5:**
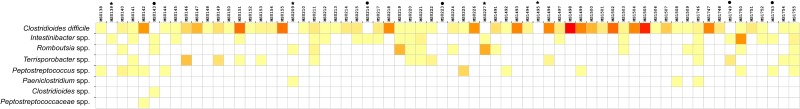
Heat map showing the distribution and relative abundance of members of the *Peptostreptococcaceae* family grouped (in rows) within the different samples (in columns). *C. difficile* was absent from nine samples: asterisks indicate the negative samples of cluster A and black dots the negative samples of cluster B.

**FIGURE 6:**
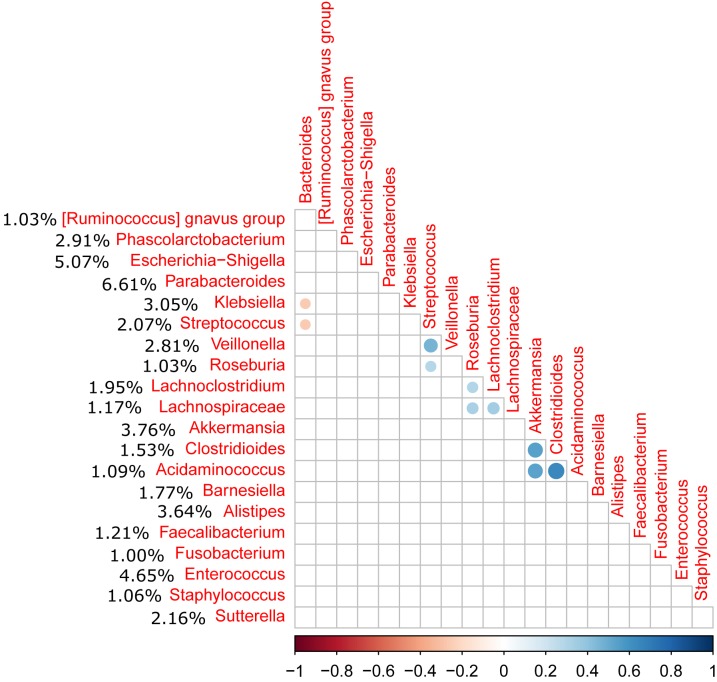
Correlation matrix of the relative abundance of genera above 1% abundance (significance level = 0.05). The average percentage of each genus among all samples is shown to the left of the name of the genus. A positive correlation was observed between the *Akkermansia* and *Clostridioides* genera (*C. difficile*).

Relative abundance of *Peptostreptococcaceae* family within the order *Clostridiales* was comparatively low, 1.56% overall, and *C. difficile* was the most abundant species within both the order *Clostridiales* (Figure 4) and the family *Peptostreptococcaceae* (Figure 5). *C. difficile* was identified in almost all samples and was detected in all but five diarrheic samples included in Cluster B (11.90% of the samples in cluster B), in which other components of the family *Peptostreptococcaceae* were found (not in sample MS0223). We detected *C. difficile* in 73.33% of the samples of cluster A. Interestingly, there was a positive correlation between the increase in the abundance of *Akkermansia* and that of the *Clostridioides* genus, as it includes only *Clostridioides difficile* (Figure 6).

## Discussion

Here, we sought to better understand the microbiota potentially associated with CDI by investigating phylogenetic variation across fecal samples from hospitalized individuals and those living in a community setting with diarrhea and a positive diagnosis of *C. difficile* by high throughput sequencing of the 16S ribosomal-RNA- gene amplicons in the Microbiology Laboratory of a tertiary hospital in Spain. Many studies have characterized the baseline gut microbiota in healthy adults, in which 90% is composed of anaerobes. Individuals can be classified into three “enterotypes” based on their microbiota composition, with a predominance of *Bacteroides*, *Prevotella*, or *Ruminococcus* genera (Arumugam et al., 2011). The individuals in this study clearly belonged to enterotype 1, with a high abundance of *Bacteroides* (31.01% overall). Many studies of the human intestinal tract have reported few phyla to be present in the gut; *Bacteroidetes* and *Firmicutes* generally dominate, whereas *Actinobacteria*, *Proteobacteria*, and *Verrucomicrobia*, are generally minor constituents. We found a higher average abundance of *Bacteroidetes* (46.51% in the samples) than in previous studies, followed by 34.70% for *Firmicutes* and 13.49% for *Proteobacteria*. However, *Firmicutes* was more prevalent in 13 samples and *Proteobacteria* in three.

Culture-based studies suggest that all healthy adults share most of the same gut bacterial species, whereas culture-independent sequencing studies have revealed vast microbial diversity (more than 1,000 species), that varies highly over time and among the population. A change in microbiota composition and a decrease in the richness of bacterial species within individuals with diarrhea and a positive diagnosis of *C. difficile* is to be expected because, although the gut microbiota stabilizes early in life (during the first 3 years), severe interventions, such as antibiotic administration, or diseases can lead to dysbiosis. Decreased diversity of the fecal microbiome has been reported in recurrent *Clostridioides difficile*-associated diarrhea. Although one of the main alpha diversity indices, the Shannon index, was significantly higher in our study than that reported in other studies (Staley et al., 2018), the findings of our study are in accordance on that decreased diversity, as we observed reduced bacterial diversity; 3,477 different ASVs were identified with high variability between samples and 29 harbored less than 100 different ASVs. Such a reduction of diversity may be associated with the bacterial dysbiosis linked to CDI. The richness of the microbiota composition was also reduced; although the microbiota composition associated with CDI is still unclear, we observed limited variability and a microbiota characteristic of the presence of *C. difficile*, independent of the severity of the disease. Interestingly, the Shannon index was very low in sample MS209, for which most of the reads were assigned to *Enterococcaceae*.; this inpatient was immunocompromised and aggressive therapy, including broad-spectrum antimicrobials, corticoids, and antifungal medicines, was used.

*Bacteroidaceae* was the most abundant family along all samples, followed by *Enterobacteriaceae*. Several families among the most abundant, such as *Lachnospiraceae* and *Ruminococcaceae*, have been previously reported to be enriched in non-diarrheal cases because they are primary butyrate-producing bacteria in the human gastrointestinal tract and have been associated with the inhibition of *C. difficile* (Schubert et al., 2014). However, the percentage of *Bacteroides* was lower among the most affected patients and in those with the worst evolution; a reduction in the abundance of this family to below 50% could be considered to be a marker for worsening of the clinical prognosis. Patients with an abnormally low abundance of *Bacteroides* (<10%) did not recover as well as those with higher percentages. We observed a reduction in the abundance of *Bacteroides* not only in *C. difficile* associated diarrhea, but also when other pathogenic agents were more abundant. This was true, for example, for samples MS1496 and MS1746, which came from the same patient diagnosed twice, 2 months apart. There was a high abundance of *Rikenellaceae* (13.65 and 15.05%), *Bacteroidaceae* was reduced to approximately 30%, and *C. difficile* was also identified in the two samples. Thus, the involvement of the pathobiont *Rikenellaceae* as a triggering factor for the diarrhea cannot be ruled out, as the percentage of *C. difficile* was lower after 2 months.

We identified two clusters in the PCoA (Figure 2); *Enterobacteriaceae* and *Enterococcaceae* were the most abundant families in cluster A, whereas *Bacteroidaceae* and *Lachnospiraceae* were the most abundant in cluster B. In addition to the different profiles of the bacterial communities, the previous medical interventions, prognoses, and recurrences were different between the two groups. Patients in cluster A were either immunosuppressed (transplant patients) or immunocompromised (cancer patients) and treated with severe antibiotic regimes, including broad spectrum antibiotics. The clinical symptoms within this group included severe diarrhea and an uncertain prognosis, which in many cases was fatal, and the percentage of recurrences higher. Patients in cluster B had had a history of less aggressive antibiotherapy, less severe diarrhea, and only a small incidence of recurrence. The use of aggressive antibiotherapy with broad spectrum antibiotics may have reduced the abundance of *Bacteroidaceae* and *Lachnospiraceae* in cluster A, giving an advantage to *Enterobacteriaceae* and *Enterococcaceae*. The clusters showed a significant difference in the level of *C. difficile* by 16S rRNA high throughput sequencing; the percentage of samples negative for *C. difficile* was 26.67% for cluster A, whereas it was only 11.90% for cluster B, for which other members of *Peptostreptococcaceae* were found in 80%. Thus, the samples belonging to cluster A likely represent a community associated with immune suppression and relatively severe antibiochemoterapy, for which the clinical prognosis of the patients is uncertain and the possibility of recurrence high. Cluster B, consisting of a compact group (Figure 2), and representing most of the samples (79.24% of the patients) may represent the general CDI scenario.

Differences in microbial composition have been already observed in CDI studies. Sangster et al. (2016) characterized 24 patients, of whom 12 were suspected of having an initial episode of CDI (not recurrent CDI). The *Lachnospiraceae*, *Bacteroidaceae*, and *Ruminococcaceae* families were dominant in both cohorts, but CDI patients showed a predominance of the *Peptostreptococcaceae* family, with a relative reduction in the abundance of the Bacteroidales and Clostridiales groups, whereas there was a higher abundance of *Akkermansia muciniphila* and some species of *Enterobacteriaceae*. Schubert et al. observed that non-diarrheal controls were more likely to have higher levels of several *Bacteroidacae, Lachnospiraceae*, and *Ruminococcaceae* families, commonly associated with a healthy microbiome and that *Enterococcus* species, *Enterobacteriaceae*, *Erysipelotrichaceae*, and some *Lachnospiraceae* families were enriched in some cases (Schubert et al., 2014). However, our findings showed *Bacteroidaceae*, *Lachnospiraceae*, and *Enterobacteriaceae* to be the most abundant families, along with a higher abundance of *Enterococcaceae*. Segregation of the samples into clusters, showed differences, as *Bacteroidaceae* and *Lachnospiraceae* were the most abundant families in cluster B (potentially associated to less severe CDI), whereas *Enterobacteriaceae* and *Enterococcaceae* were the most abundant in Cluster A, potentially associated with immune suppression, previous use of aggressive antibiotherapy, severe diarrhea, and a high incidence of recurrence.

In a recent study of more than 80 patients, the most abundant bacterial family was *Enterobacteriaceae* (>30%) and the five most abundant families (*Enterobacteriaceae*, *Lachnospiraceae*, *Bacteroidaceae, Porphyromonadaceae*, and *Ruminococcaceae*) represented less than 50% of the bacterial diversity (Staley et al., 2018). In our study, *Enterobacteriaceae* was the most abundant family in cluster A (22.66% overall, but ranged from 0% in sample MS1495 to 79.13% in sample MS1508), whereas the relative abundance of *Lachnospiraceae* was significantly lower (4.96% overall). Staley et al. (2018) also reported similar figures; *Enterobacteriaceae* was the most abundant bacterial family and the abundance of *Lachnospiraceae* was also very low. However, *Lachnospiraceae* was the third most abundant family overall (9.33%) and the second most abundant family in cluster B, in which its relative abundance was even higher (10.64%). This finding has been previously reported, but to a significantly lower extent: i.e., 6.5% (Staley et al., 2018). Although *Lachnospiraceae* are common inhabitants of the healthy human and mammalian gut microbiota (Lagier et al., 2012), they are highly sensitive to antimicrobial chemotherapy. Thus, a common sign of antibiotic exposure is the reduction or absence of *Lachnospiraceae* in the gut, creating an environmental niche for opportunistic CDI (Song et al., 2013). Not surprisingly, restoring *Lachnospiraceae* in infected patients has been shown to help cure *C. difficile* infections (Song et al., 2013) and it is a bacterial family used in fecal material transplants (Staley et al., 2018). Similarly, a member of the *Tannerellaceae* family, *Parabacteroides distasonis*, has been reported to be the most abundant in certain gastrointestinal disorders, such as Crohn’s disease (Lopetuso et al., 2018). Sample MS0147 came from a patient with Crohn’s disease and the most abundant genus was Escherichia–Shigella at 22.65%.

Although the relative abundance of *Peptostreptococcaceae* was comparatively low (1.56% overall), this family is of prime importance in CDI. The clostridial phylogeny in the phylum *Firmicutes* has recently been reconsidered and, among other reassignments of the taxa, it has been suggested that *C. difficile* and its close relatives, *C. paradoxum* and *C. sticklandii*, be reclassified within the family *Peptostreptococcaceae* in the order *Clostridiales* as a tentative solution to resolve various taxonomical problems (Yutin and Galperin, 2013). As expected, *C. difficile* was the most abundant species within both the order *Clostridiales* and the family *Peptostreptococcaceae*, and was identified in almost all samples. We also observed a correlation between the presence of *Akkermansia* and *Peptostreptococcaceae* as previously reported (Sangster et al., 2016). Sangster et al. (2016) also reported an increase in the abundance of *Akkermansia muciniphila* in CDI patients, potentially linked to the capacity of *Akkermansia* to degrade mucin, which may provide a selective advantage in CDI. In our study, samples MS0223 and MS1753 exhibited an abnormal expansion of the family *Prevotellaceae* (16.50% and 32.25%, respectively). *Prevotellaceae* is a family that can enzymatically disrupt mucosal barrier function and tends to be more abundant in intestinal biopsy samples isolated from patients with inflammatory bowel disease (IBD) (Nagao-Kitamoto et al., 2016).

Predicting microbiota dynamics in individuals and determining whether changes in composition are associated with varying severity and outcomes of CDI will require determining whether such changes lead to the disease. Wilson and Perini (1988) and latter other authors, demonstrated that other microorganisms compete more efficiently than *C. difficile* for monomeric glucose, *N*-acetylglucosamine, and sialic acids found in colonic contents, being the reason why *C. difficile* is not a predominant intestinal bacterium. However, when perturbations of the gut microbiota, as occur upon antibiotic administration, intestinal commensal bacteria depletion occur and consequently can help *C. difficile* compete for nutrients to grow, as well as fermentation process decline causing an increase of fecal redox potential. We analyzed the redox potential in fecal content and differs among the samples (minimum +29.03 mV, maximum +187,67 mV, mean ± standard deviation, 90.7 mV ± SD35.1). While we tried to establish a correlation between gut redox modification and the microbial alterations observed, it remains unclear, although our findings could suggest that modifications of the redox potential might be a key parameter shaping the gut microbiota to be elucidated in future studies. On the other hand, virulence, previous medical treatment, and host immunity will also need to be considered. We analyzed only samples from patients with diarrhea and a positive diagnosis for *C. difficile*. However, several other aspects must be considered in the association of the microbiota to CDI and other gastrointestinal processes. First, a potential drawback of ecological studies of gut microbiota in pathological processes is the misdiagnosis and/or the misassumption of the role of *C. difficile* in the gastrointestinal process, as patients colonized by *C. difficile* can be found for whom the diarrhea is primarily caused by other pathogens, although *C. difficile* is present. That was true in our study for at least four patients who were infected by *Klebsiella*, *Campylobacter*, *Staphylococcus*, or *Escherichia*. In these cases, co-infection cannot be ruled out, and the differences in the bacterial community profile should be considered with caution. The previous history of antibiotherapy is also highly relevant; the trigger may be dysbiosis caused by prior antibiotic treatment, even in cases in which the gastrointestinal process is caused by *C. difficile* and there is robust evidence for CDI. Similarly, the trigger may be also the status of the immune system. In our study, we were able to segregate the samples into clusters in which these two aspects were highly relevant. Finally, we cannot rule out that the triggering factors were digestive disorders or gastrointestinal diseases (small bowel occlusion, Chron’s disease, gastritis, diverticulitis).

In conclusion, our findings show that a reduction in *Bacteroides* is a clear disadvantage for healthy gut microbiota and can result in a worse CDI prognosis, including severe diarrhea and a high incidence of recurrence. This reduction may be associated with a weakened host immune system and history of aggressive antibiotherapy. In addition, an elevated abundance of *Akkermansia* may be a predictive marker for the presence of a CDI. Finally, a relevant aspect that must be considered in clinical practice is the misdiagnosis of CDI, as patients with a stool sample that tests positive for *C. difficile* are usually diagnosed with CDI and subsequently treated as such. However, co-infection with other pathogenic agents often plays an important role in the development of diarrhea and must be considered when prescribing antibiotic treatment.

### Nucleotide Sequence Accession Number

The 16S rRNA profiling data sequenced in this study were deposited in the Sequence Read Archive of the National Center for Biotechnology Information database under the following study accession number: PRJNA493204.

## Author Contributions

MF, MH, and JE designed the study. MF and LL-U performed the diagnosis experiments. MH performed the sequencing experiments. MH and NQ performed the bioinformatics analysis and designed the figures. MH and JE drafted the manuscript. DR-L contributed to the discussion of the results and to the writing of the manuscript.

## Conflict of Interest Statement

The authors declare that the research was conducted in the absence of any commercial or financial relationships that could be construed as a potential conflict of interest.

## Footnotes

1 www.cdc.gov/media/releases/2015/p0225-clostridium-difficile.html

2 https://ecdc.europa.eu/sites/portal/files/media/en/publications/Publications/healthcare-associated-infections-antimicrobial-use-PPS.pdf

3 https://github.com/qiime2/q2-diversity

4 https://www.r-project.org

## References

[B1] Alcalá L. Reigadas E. Marín M. Martín A. Catalán P. Bouza E. (2015). Impact of clinical awareness and diagnostic tests on the underdiagnosis of *Clostridium difficile* infection. *Eur. J. Clin. Microbiol. Infect. Dis.* 34 1515–1525. 10.1007/s10096-015-2380-3 25904126

[B2] Arumugam M. Raes J. Bork P. (2011). Enterotypes of the human gut microbiome. *Nature* 473 174–180. 10.1038/nature09944 21508958 PMC3728647

[B3] Bokulich N. A. Kaehler B. D. Rideout J. R. Dillon M. Bolyen E. Knight R. (2018). Optimizing taxonomic classification of marker-gene amplicon sequences with qiime 2’s q2-feature-classifier plugin. *Microbiome* 6:90. 10.1186/s40168-018-0470-z 29773078 PMC5956843

[B4] Callahan B. J. McMurdie P. J. Holmes S. P. (2017). Exact sequence variants should replace operational taxonomic units in marker-gene data analysis. *ISME J.* 11 2639–2643. 10.1038/ismej.2017.119 28731476 PMC5702726

[B5] Callahan B. J. McMurdie P. J. Rosen M. J. Han A. W. Johnson A. J. A. Holmes S. P. (2016). DADA2: high-resolution sample inference from Illumina amplicon data. *Nat. Methods* 13 581–583. 10.1038/nmeth.3869 27214047 PMC4927377

[B6] Caporaso J. G. Kuczynski J. Stombaugh J. Bittinger K. Bushman F. D. Costello E. K. (2010). QIIME allows analysis of high-throughput community sequencing data. *Nat. Methods* 7 335–336. 20383131 10.1038/nmeth.f.303 PMC3156573

[B7] Davies K. A. Ashwin H. Longshaw C. M. Burns D. A. Davis G. L. Wilcox M. H. (2016). Diversity of *Clostridium difficile* PCR ribotypes in Europe: results from the European, multicentre, prospective, biannual, point-prevalence study of *Clostridium difficile* infection in hospitalised patients with diarrhoea (EUCLID), 2012 and 2013. *Euro Surveill.* 21:30294. 10.2807/1560-7917.ES.2016.21.29.30294 27470194

[B8] Davies K. A. Longshaw C. M. Davis G. L. Bouza E. Barbut F. Barna Z. (2014). Underdiagnosis of *Clostridium difficile* across Europe: the European, multicentre, prospective, biannual, point-prevalence study of *Clostridium difficile* infection in hospitalised patients with diarrhea (EUCLID). *Lancet Infect. Dis.* 14 1208–1219. 10.1016/S1473-3099(14)70991-0 25455988

[B9] Di Bella S. Ascenzi P. Siarakas S. Petrosillo N. di Masi A. (2016). *Clostridium difficile* toxins A and B: insights into pathogenic properties and extraintestinal effects. *Toxins* 8:E134. 10.3390/toxins8050134 27153087 PMC4885049

[B10] Hensgens M. P. M. Goorhuis A. Dekkers O. M. van Benthem B. H. B. Kuijper E. J. (2013). All-cause and disease-specific mortality in hospitalized patients with *Clostridium difficile* infection: a multicenter cohort study. *Clin. Infect. Dis.* 56 1108–1116. 10.1093/cid/cis1209 23300235

[B11] Joachimiak M. P. Weisman J. L. May B. (2006). JColorGrid: software for the visualization of biological measurements. *BMC Bioinformatics* 7:225. 10.1186/1471-2105-7-225 16640789 PMC1479842

[B12] Juul F. E. Garborg K. Bretthauer M. Skudal H. Øines M. N. Wiig H. (2018). Fecal microbiota transplantation for primary *Clostridium difficile* infection. *N. Engl. J. Med.* 378 2535–2536. 10.1056/NEJMc1803103 29860912

[B13] Katoh K. Standley D. M. (2013). MAFFT multiple sequence alignment software version 7: improvements in performance and usability. *Mol. Biol. Evol.* 30 772–780. 10.1093/molbev/mst010 23329690 PMC3603318

[B14] Klindworth A. Pruesse E. Schweer T. Peplies J. Quast C. Horn M. (2013). Evaluation of general 16S ribosomal RNA gene PCR primers for classical and next-generation sequencing-based diversity studies. *Nucleic Acids Res.* 41:e1. 10.1093/nar/gks808 22933715 PMC3592464

[B15] Knight D. R. Elliott B. Chang B. J. Perkins T. T. Riley T. V. (2015). Diversity and evolution in the genome of *Clostridium difficile*. *Clin. Microbiol. Rev.* 28 721–741. 10.1128/CMR.00127-14 26085550 PMC4475645

[B16] Lagier J.-C. (2016). Gut microbiota and *Clostridium difficile* infections. *Hum. Microbiome J.* 2 10–14. 10.1016/j.humic.2016.10.003

[B17] Lagier J.-C. Million M. Hugon P. Armougom F. Raoult D. (2012). Human gut microbiota: repertoire and variations. *Front. Cell. Infect. Microbiol.* 2:136 10.3389/fcimb.2012.00136 PMC3487222 23130351

[B18] Lees E. A. Miyajima F. Pirmohamed M. Carrol E. D. (2016). The role of *Clostridium difficile* in the paediatric and neonatal gut — a narrative review. *Eur. J. Clin. Microbiol. Infect. Dis.* 35 1047–1057. 10.1007/s10096-016-2639-3 27107991 PMC4902830

[B19] Leffler D. A. Lamont J. T. (2015). *Clostridium difficile* infection. *N. Engl. J. Med.* 372 1539–1548. 25875259 10.1056/NEJMra1403772

[B20] Lopetuso L. R. Petito V. Graziani C. Schiavoni E. Paroni Sterbini F. Poscia A. (2018). Gut microbiota in health, diverticular disease, irritable bowel syndrome, and inflammatory bowel diseases: time for microbial marker of gastrointestinal disorders. *Dig. Dis.* 36 56–65. 10.1159/000477205 28683448

[B21] McDonald D. Price M. N. Goodrich J. Nawrocki E. P. DeSantis T. Z. Probst A. (2012). An improved greengenes taxonomy with explicit ranks for ecological and evolutionary analyses of bacteria and archaea. *ISME. J.* 6 610–618. 10.1038/ismej.2011.139 22134646 PMC3280142

[B22] McFarland L. V. Mulligan M. E. Kwok R. Y. Stamm W. E. (1989). Nosocomial acquisition of *Clostridium difficile* infection. *N. Engl. J. Med.* 320 204–210. 2911306 10.1056/NEJM198901263200402

[B23] Nagao-Kitamoto H. Kitamoto S. Kuffa P. Kamada N. (2016). Pathogenic role of the gut microbiota in gastrointestinal diseases. *Intest. Res.* 14 127–138. 10.5217/ir.2016.14.2.127 27175113 PMC4863046

[B24] Price M. N. Dehal P. S. Arkin A. P. (2010). FastTree 2–approximately maximum-likelihood trees for large alignments. *PLoS One* 5:e9490. 10.1371/journal.pone.0009490 20224823 PMC2835736

[B25] Pruesse E. Quast C. Knittel K. Fuchs B. M. Ludwig W. Peplies J. (2007). SILVA: a comprehensive online resource for quality checked and aligned ribosomal RNA sequence data compatible with ARB. *Nucleic Acids Res.* 35 7188–7196. 17947321 10.1093/nar/gkm864 PMC2175337

[B26] Rupnik M. (2007). Is *Clostridium difficile*-associated infection a potentially zoonotic and foodborne disease? *Clin. Microbiol. Infect.* 13 457–459. 17331126 10.1111/j.1469-0691.2007.01687.x

[B27] Sangster W. Hegarty J. P. Schieffer K. M. Wright J. R. Hackman J. Toole D. R. (2016). Bacterial and fungal microbiota changes distinguish *C. difficile* infection from other forms of diarrhea: results of a prospective inpatient study. *Front Microbiol.* 25:789. 10.3389/fmicb.2016.00789 27252696 PMC4879479

[B28] Schubert A. M. Rogers M. A. M. Ring C. Mogle J. Petrosino J. P. Young V. B. (2014). Microbiome data distinguish patients with *Clostridium difficile* infection and non-*C. difficile*-associated diarrhea from healthy controls. *mBio* 5:e01021-14. 10.1128/mBio.01021-14 24803517 PMC4010826

[B29] Sebaihia M. Wren B. W. Mullany P. Fairweather N. F. Minton N. Stabler R. (2006). The multidrug-resistant human pathogen *Clostridium difficile* has a highly mobile, mosaic genome. *Nat. Genet.* 38 779–786. 16804543 10.1038/ng1830

[B30] Shankar V. Hamilton M. J. Khoruts A. Kilburn A. Unno T. Paliy O. (2014). Species and genus level resolution analysis of gut microbiota in *Clostridium difficile* patients following fecal microbiota transplantation. *Microbiome* 2:13. 10.1186/2049-2618-2-13 24855561 PMC4030581

[B31] Smits W. K. Lyras D. Lacy D. B. Wilcox M. H. Kuijper E. J. (2016). *Clostridium difficile* infection. *Nat. Rev. Dis. Primers* 7:16020. 10.1038/nrdp.2016.20 27158839 PMC5453186

[B32] Song Y. Garg S. Girotra M. Maddox C. von Rosenvinge E. C. Dutta A. (2013). Microbiota dynamics in patients treated with fecal microbiota transplantation for recurrent *Clostridium difficile* infection. *PLoS One* 8:e81330. 10.1371/journal.pone.0081330 24303043 PMC3841263

[B33] Staley C. Kaiser T. Vaughn B. P. Graiziger C. T. Hamilton M. J. Rehman T. U. (2018). Predicting recurrence of *Clostridium difficile* infection following encapsulated fecal microbiota transplantation. *Microbiome* 6:166. 10.1186/s40168-018-0549-6 30227892 PMC6145197

[B34] Vazquez-Baeza Y. Pirrung M. Gonzalez A. Knight R. (2013). EMPeror: a tool for visualizing high-throughput microbial community data. *Gigascience* 2:16. 10.1186/2047-217X-2-16 24280061 PMC4076506

[B35] Wickham H. (2007). Reshaping data with the reshape package. *J. Stat. Softw.* 21 1–20. 10.3978/j.issn.2305-5839.2016.01.33 27004225 PMC4779770

[B36] Wickham H. (2016). *ggplot2: Elegant Graphics for Data Analysis*. New York, NY: Springer-Verlag.

[B37] Wilson K. H. Perini F. (1988). Role of competition for nutrients in suppression of Clostridium difficile by the colonic microflora. *Infect. Immun.* 56 2610–2614. 3417352 10.1128/iai.56.10.2610-2614.1988 PMC259619

[B38] Yutin N. Galperin M. Y. (2013). A genomic update on clostridial phylogeny: Gram-negative spore formers and other misplaced clostridia. *Environ. Microbiol.* 15 2631–2641. 10.1111/1462-2920.12173 23834245 PMC4056668

